# Distinct acute stressors produce different intensity of anxiety-like behavior and differential glutamate release in zebrafish brain

**DOI:** 10.3389/fnbeh.2024.1464992

**Published:** 2024-10-23

**Authors:** Milena Letícia Martins, Emerson Feio Pinheiro, Geovanna Ayami Saito, Caroline Araújo Costa De Lima, Luana Ketlen Reis Leão, Evander de Jesus Oliveira Batista, Adelaide da Conceição Fonseca Passos, Amauri Gouveia, Karen Renata Herculano Matos Oliveira, Anderson Manoel Herculano

**Affiliations:** ^1^Laboratory of Experimental Neuropharmacology, Biological Science Institute, UFPA, Belém, Brazil; ^2^Laboratory of Protozoology, Tropical Medicine Nucleus, UFPA, Belém, Brazil; ^3^Laboratory of Neuroscience and Behavior, UFPA, Belém, Brazil

**Keywords:** anxiety-like behavior, stress, neurochemistry, glutamate, zebrafish

## Abstract

Anxiety disorder is one of the most well-characterized behavioral disorders in individuals subjected to acute or chronic stress. However, few studies have demonstrated how different types of stressors can modulate the neurochemical alterations involved in the generation of anxiety. In this study, we hypothesize that subjects exposed to different aversive stimuli (mechanical, chemical, and spatial restriction) present varied intensities of anxiety-like responses associated with distinct patterns of gamma-aminobutyric acid (GABA) and glutamate release in the brain. Adult zebrafish, *Danio rerio* (*n* = 60), were randomly divided into four experimental groups; control, acute restraint stress (ARS), conspecific alarm substance (CAS), and chasing with net (CN). After the stress protocols, the animals were individually transferred to a novel tank diving test for behavioral analysis. Subsequently, their brains were collected and subjected to GABA and glutamate release assay for quantification by HPLC. Our behavioral results showed that all aversive stimuli were capable of inducing anxiety-like behavior. However, the impact of anxiogenic behavior was more prominent in the CN and CAS groups when compared to ARS. This phenomenon was evident in all analyzed behavioral parameters (time on top, freezing, mean speed, maximum speed, and erratic swimming). Our data also showed that all aversive stimuli significantly decreased GABA release compared to the control group. Only animals exposed to CN and CAS presented an increase in extracellular glutamate levels. Different acute stressors induced different levels of anxiety-like behavior in zebrafish as well as specific alterations in GABAergic and glutamatergic release in the brain. These results demonstrate the complexity of anxiety disorders, highlighting that both behavioral and neurochemical responses are highly context-dependent.

## 1 Introduction

Anxiety disorder represents one the most well characterized behavioral alterations in subjects submitted to acute or chronic stress (Egan et al., 2010; Piato et al., 2011; Etaee et al., 2019; Harada et al., 2024). As described by World Health Organization (2022), post-traumatic disorders, including anxiety disorder, affect millions of people around the world and represent a serious problem of public health in different countries. Traumatic events induce severe pattern anxiety disorder characterized by intense somatic symptoms (e.g., palpitations, dizziness, dyspnea) as well as emotional and cognitive impairments (e.g., negative affect, fear, worry, and rumination; Szuhany and Simon, 2022; Andreescu, 2015). Together, these symptoms lead many people to suicide, social isolation or retirement (Behrens-wittenberg and Wedegaertner, 2021; Nepon et al., 2011; Wolters et al., 2023. In face of these data, many efforts are focused on clarifying the brain alterations related with the development of anxiety disorder triggered by different acute stress.

It is well documented that the behavioral aspects inherent to anxiety are mediated by alterations in the homeostasis of different neurotransmitters system (Sandford et al., 2001; Millan, 2003) as well as in anatomically distinct areas of the brain (Anand and Shekhar, 2003; Robinson et al., 2019). Among them, the role of gamma-aminobutyric acid (GABA) stands out, whose inhibitory action on the limbic system decreases defense reactions against threats (Nutt and Malizia, 2001; Lydiard, 2003; Kalueff and Nutt, 2007; Nuss, 2015). Alterations in GABA levels are believed to affect the susceptibility of the CNS to the actions of glutamate, resulting in hyperactivation of systems associated with the classic symptoms of anxiety, making these neurotransmitters fundamental in the development of the disorder (Kalueff and Nutt, 2007; Wierońska, 2011; Nuss, 2015). Currently, one of the pharmacological strategies for the treatment of anxiety disorders is based on positive modulation of GABAergic receptors to restore control of the excitability of local circuits, as observed with the use of benzodiazepines (Nuss, 2015). Furthermore, due to the involvement of other neurotransmission systems, such as noradrenergic and serotonergic, antidepressants are also frequently prescribed (Fluyau et al., 2022; Lee and Stein, 2023). However, despite their efficacy, these drugs are associated with adverse effects, such as dependence, sedation, memory impairment, and drowsiness (Baldwin et al., 2013; Sinha et al., 2017). Given these limitations, there is a growing need to explore new modulators that can alter neuronal function and behavioral processes with better tolerability. Since benzodiazepines exert their anxiolytic effects through increased GABA-mediated inhibitory transmission, an alternative strategy would be to reduce glutamatergic excitatory neurotransmission (Wierońska, 2011).

Previous reports have already described that different kinds of aversive stimuli can trigger anxiety disorders (Egan et al., 2010; de Abreu et al., 2021; Harada et al., 2024). However, it remains unclear how stressors of different natures (mechanical, physical, or chemical) alter brain neurochemistry, evoking various types of anxiety disorder. In this study, we hypothesize that different aversive stimuli elicit distinct responses in the GABAergic and glutamatergic systems, associated with varying intensities of anxiety-like behavior. To test this hypothesis, we used zebrafish (*Danio rerio*), a well-established animal model for investigating the neurobiology of various neurological disorders (Panula et al., 2010; Maximino et al., 2011; Stewart et al., 2012; Fontana et al., 2020).

## 2 Materials and methods

### 2.1 Animals and housing

Sixty *Danio rerio* (zebrafish) *short-fin* from 3 to 4 months old, weighing 0.3 g (± 0.2) from both sexes, were purchased from a local supplier (Belém-Pará). Fish were acclimated in 50 L tanks (50 × 35 × 30) at 28°C ± 2, pH 7.2 ± 0.2, oxygenation, 14-h/10-h light/dark controlled photoperiod and fed once a day with commercial flocculated feed (Tetra, Germany) with density of 1 animal per liter and were acclimatized for a minimum period of 15 days, according to previous studies performed by our group. The animals were randomly divided into 4 experimental groups: Control CTRL (*n* = 15), Acute Restraint Stress ARS (*n* = 15), Conspecific Alarm Substance CAS (*n* = 15) and Chasing with Net CN (*n* = 15). All experimental procedures were made in accordance with the National Council of Animal Experimentation Control (CONCEA) and previously approved by the Committee of Ethics in Research with Experimental Animals of the Federal University of Pará (CEPAE—UFPA: 213–14).

### 2.2 Stressors protocol

The induction of stress was performed using three stressors of different natures (spatial, mechanical and chemical restriction). Spatial restriction-based stress was performed using the Acute Restraint Stress (ARS) protocol, performed as described by Piato et al. (2011), that consists of conditioning the animal in a plastic microtube (2 mL), with a small opening at both sides, to restrict its locomotion for 90 min. The mechanical stress described by De Abreu et al. (2014) is denominated Chasing with Net (CN). In this method, animals are individually relocated for an apparatus where they are subjected to circular chasing movements with a net, without capture, for 2 min. To induce chemical stress, exposure to Conspecific Alarm Substance (CAS) was conducted, as detailed by Silva et al. (2018). This protocol involves extracting the alarm substance from a donor fish. This individual was cryoanesthetized and decapitated with a scalpel, and 15 superficial cuts were made on each side of the trunk to damage the epithelial cells without causing blood leakage. The body was then transferred to another Petri dish, and the cuts were carefully washed with 10 mL of distilled water to remove impurities, preserving 7 mL as the CAS unit. This solution was then stored in the refrigerator and could be kept for up to 4 h after extraction. For exposure to chemical stress, the animals were individually transferred to a tank, to which 7 mL of CAS was added for 6 min. After this period, the animals were moved to the experimental apparatus.

### 2.3 Novel tank diving test and experimental procedure

**Figure 1:**
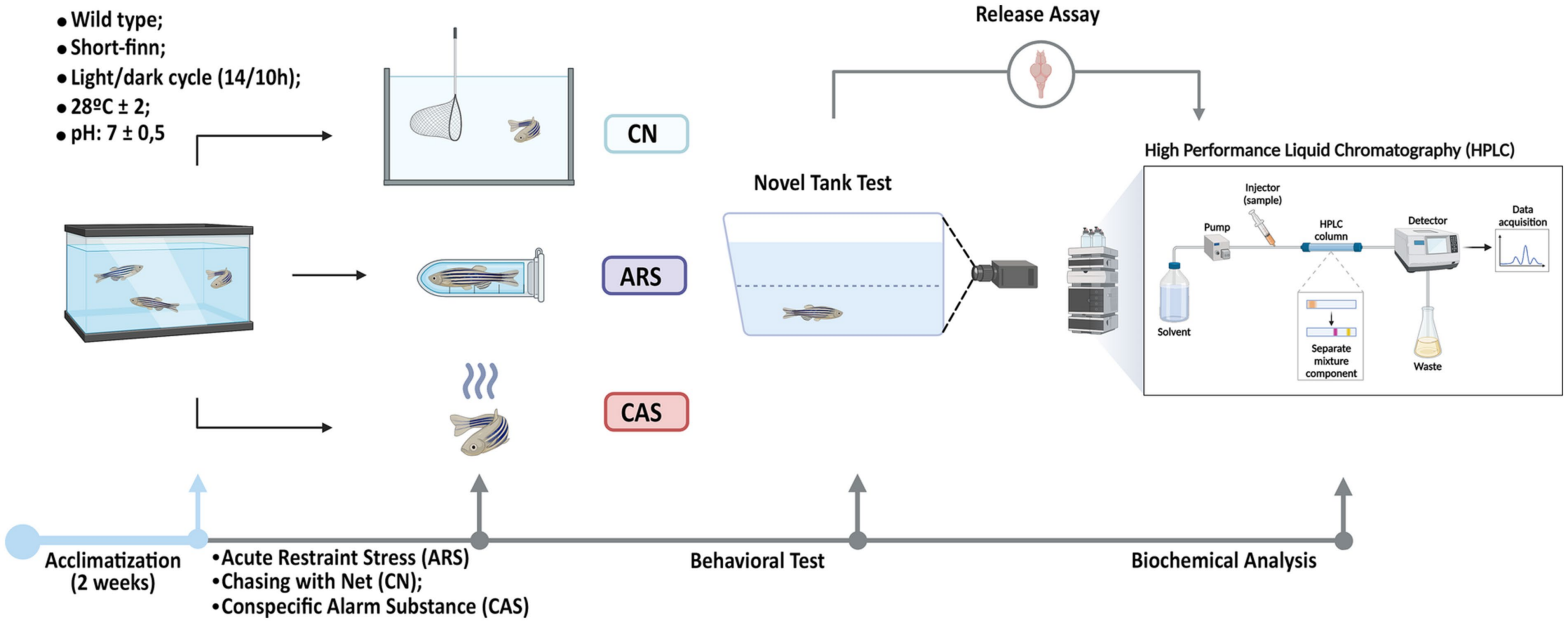
Experimental procedures. After a two-week acclimatization period, the animals were subdivided into four groups: Control (CTRL), Acute Restraint Stress (ARS), Chasing with Net (CN), and Conspecific Alarm Substance (CAS). Immediately following exposure to the aversive stimuli, the Novel Tank Diving Test was conducted, during which the following parameters were measured: Time on Top (s), Freezing (s), Mean Speed (cm/s), Erratic Swimming (f), and Maximum Speed (cm/s). Finally, after the behavioral tests, the brains of the animals were collected for biochemical assays, and the levels of GABA and glutamate were quantified using the HPLC method.

After applying the stress protocols, the animals were immediately and individually transferred to the Novel Tank Diving Test apparatus (30 cm length × 22 cm height × 15 cm width) within their respective groups. All behavioral experiments were conducted between 8:00 am and 1:00 pm, with controlled lighting under the apparatus at a mean of 500–600 lux. Each animal’s free exploration was recorded by a camera for 10 min and subsequently analyzed using ZabTrack software (Pinheiro-da-Silva, 2017) to quantify the following variables: Time on Top (s): Duration spent by the animal in the upper zone of the tank; Freezing (s): Defined as periods when the animal remains completely immobile for at least 5 s, accompanied by an increased opercular beat rate; Erratic swimming (frequency): Erratic swimming (frequency): characterized by a rapid, zigzag, and unpredictable swimming path of short duration; Mean speed (cm/s): mean speed of the animal’s movement during observation; Maximum speed (cm/s): highest speed recorded during the exploration period (Kalueff et al., 2013). Following the behavioral assessment, the animals were euthanized, and their brains were dissected for a biochemical assay to quantify the neurotransmitters GABA and glutamate using the HPLC method (Figure 1).

### 2.4 Neurotransmitter release assay and quantification by HPLC

After behavioral evaluation, the animals were euthanized and the brain was dissected and submitted to the glutamate and GABA release assay, as previously described by Moraes et al. (2012). Samples were rinsed three times with Hank Balanced Salt Solution (HBSS) consisting of in (mM): NaCl 128, KCl 4, MgCl 2, CaCl2, glucose 12 and HEPES 20, pH 7.2–7.4 adjusted with NaOH 1 N. The samples were incubated with 350 μL of HBSS at 37°C in a CO_2_ incubator for 20 min. Subsequently, GABA and glutamate levels were quantified using High-performance Liquid Chromatography (Shimadzu LC-10 AD) equipped with a C18 analytical column (Shim-pack VP-ODS 4.6*250LC, internal diameter 4.6 mm), coupled to a fluorescence detector (RF-10AXL). All reagents used were of high purity, purchased from Sigma-Aldrich, and the dilution water was Milli-Q deionized. The quantification values of GABA and Glutamate release were expressed as a percentage of the control and the protein dosage was performed using the Bradford (1976) method. Gaba and glutamate levels were measured at μM/mg/min obtaining the following values—GABA: CTRL = 4.785 ± 1.766; ARS = 1.419 ± 0.3424; CN = 1.34 ± 0.3914 and CAS = 1.521 ± 0.4311; glutamate: CTRL = 1.751 ± 0.5598; ARS = 2.201 ± 0.6482; CN = 3.320 ± 0.7283 and CAS = 3.201 ± 1.101.

### 2.5 Statistical analysis

Data are presented as median and the percentiles minimum and maximum are represented by the whiskers for behavioral analysis and percentage of controls for biochemical analysis. The normal distribution of data was determined by the Shapiro–Wilk test. One-way analysis of variance (ANOVA) followed by Tukey’s *post-hoc* test was applied to evaluate the biochemical and behavioral data. Linear regression analysis was used to assess the association between time spent on top and GABA and glutamate levels for the control, ARS, CN, and CAS groups. All analyzes were performed using the GraphPad Prism software version 10.0.0 (GraphPad Software Inc., San Diego, CA, United States), with a significance level of *p* < 0.05.

## 3 Results

### 3.1 Evaluation of anxiety-like behavior induced by ARS, CN and CAS

**Figure 2:**
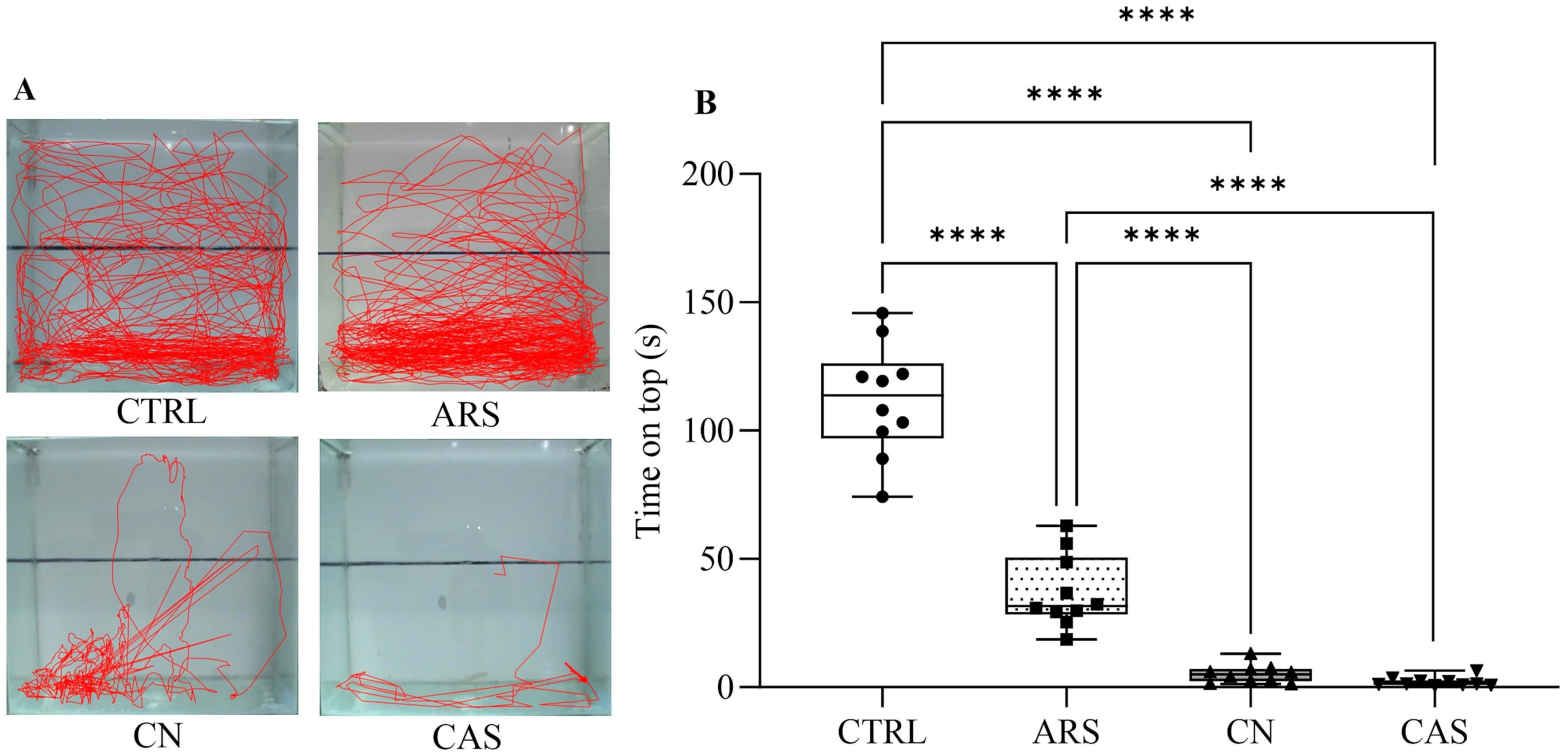
Effect of stressors on animal trajectory (A) and box plot demonstrating the time spent on top (B) during Novel Tank Diving test. Results were expressed as median and the percentiles minimum and maximum represented by the whiskers. Data were compared using one-way ANOVA followed by Tukey’s *post-hoc* test. ****p < 0.0001, ***p < 0.001, **p < 0.01, *p < 0.05. CTRL, control; ARS, Acute Restraint Stress; CN, Chasing with Net; CAS, Conspecific Alarm Substance.

**Figure 3:**
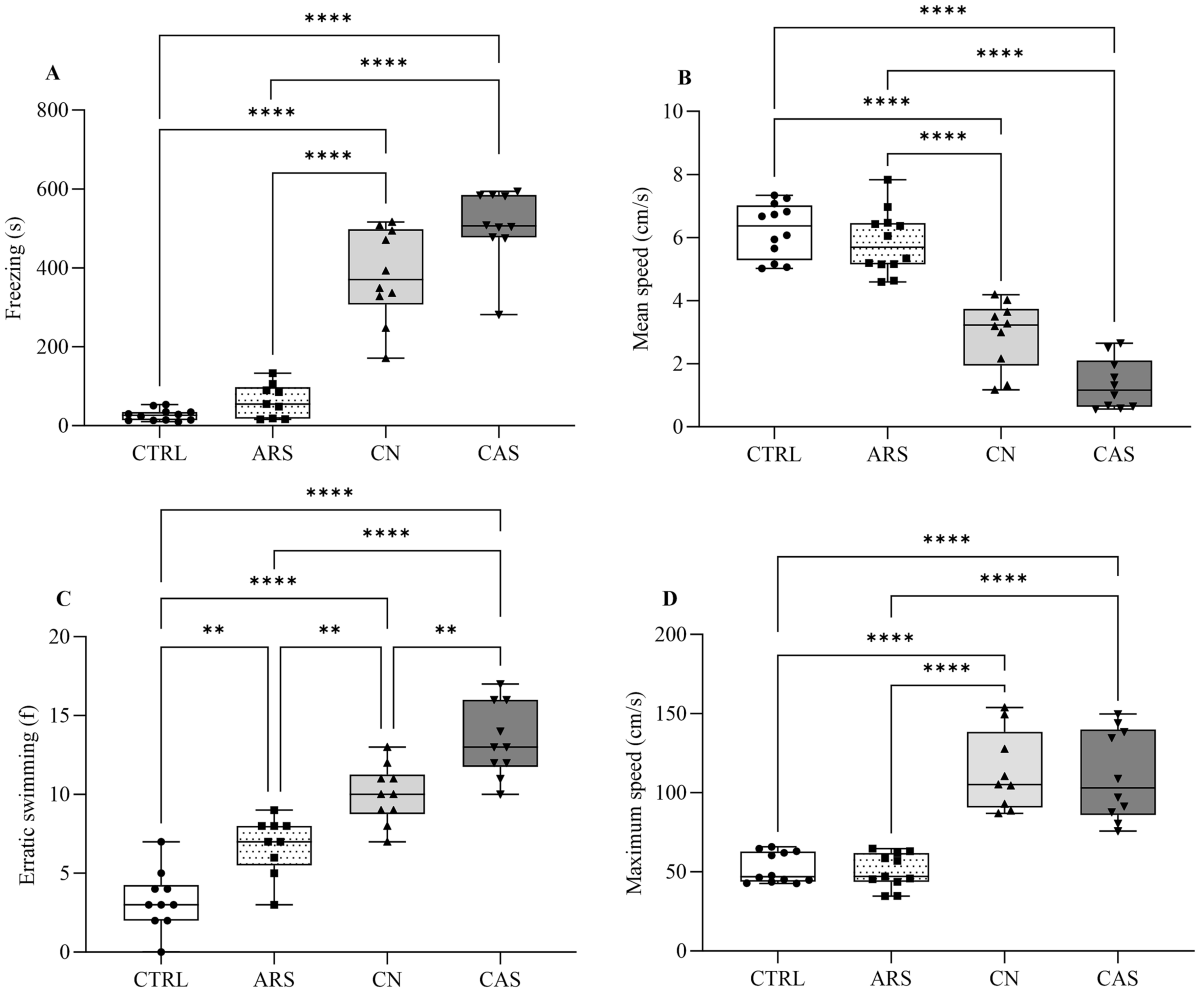
Box plot demonstrating the effect of aversive stimuli during Novel Tank Diving test on behavioral locomotion evaluated by the parameters: Freezing (A) and Erratic swimming (B); Mean speed (C) and Maximum speed (D). The results were expressed as median and the percentiles minimum and maximum are represented by the whiskers. Data were compared using ANOVA one-way followed by Tukey *post-hoc* test. ****p < 0.0001, ***p < 0.001, **p < 0.01, *p < 0.05. CTRL, control; ARS, Acute Restraint Stress; CN, Chasing with Net; CAS, Conspecific Alarm Substance.

In order to evaluate the effect of different stressors on the anxiety-like behavior in zebrafish, the subjects were acclimatized and then submitted to chasing with net (CN), acute restraint stress (ARS) and conspecific alarm substance (CAS) as described in Figure 1. The results of the Novel Tank Diving test demonstrated that all aversive stimuli induced different phenotypes of anxiety-like behavior in zebrafish. In Figure 2A, we show the different patterns of exploratory behavior in the Novel Tank Diving test for the CTRL, ARS, CN, and CAS groups. When analyzing these patterns, we observed a significant reduction in the time spent on top of the tank in the groups exposed to the stimuli compared to the CTRL group (*F*_(3, 36)_ = 150.7; *p* < 0.0001; Figure 2B). Our results demonstrated that the reduction in time spent at the top of the tank was more pronounced in the groups exposed to the alarm substance and chasing with net, compared to those exposed to acute restraint stress (F _(3, 36)_ = 150.7; *p* < 0.0001; Figure 2B). Additionally, we observed that exposure to the CN and CAS protocols promoted an increase in freezing time of the animals compared to the CTRL and ARS groups (*F*_(3, 37)_ = 99.27; *p* < 0.0001; Figure 3A). These data justify the significant decrease in mean speed indices in CN and CAS groups compared to CTRL and ARS groups (*F*_(3, 40)_ = 68.21; *p* < 0.0001; Figure 3B). Based on the erratic swimming parameter, anxiogenic behavior was more pronounced in the CN and CAS groups compared to the CTRL and ARS groups (*F*_(3, 35)_ = 47.27; *p* < 0.0001; Figure 3C). This parameter possible influenced the maximum speed indices of the CN and CAS groups compared to the CTRL and ARS groups (*F*_(3, 41)_ = 26.12; *p* < 0.0001; Figure 3D).

### 3.2 GABA and glutamate quantification in the brain of zebrafish submitted to ARS, CN and CAS

**Figure 4:**
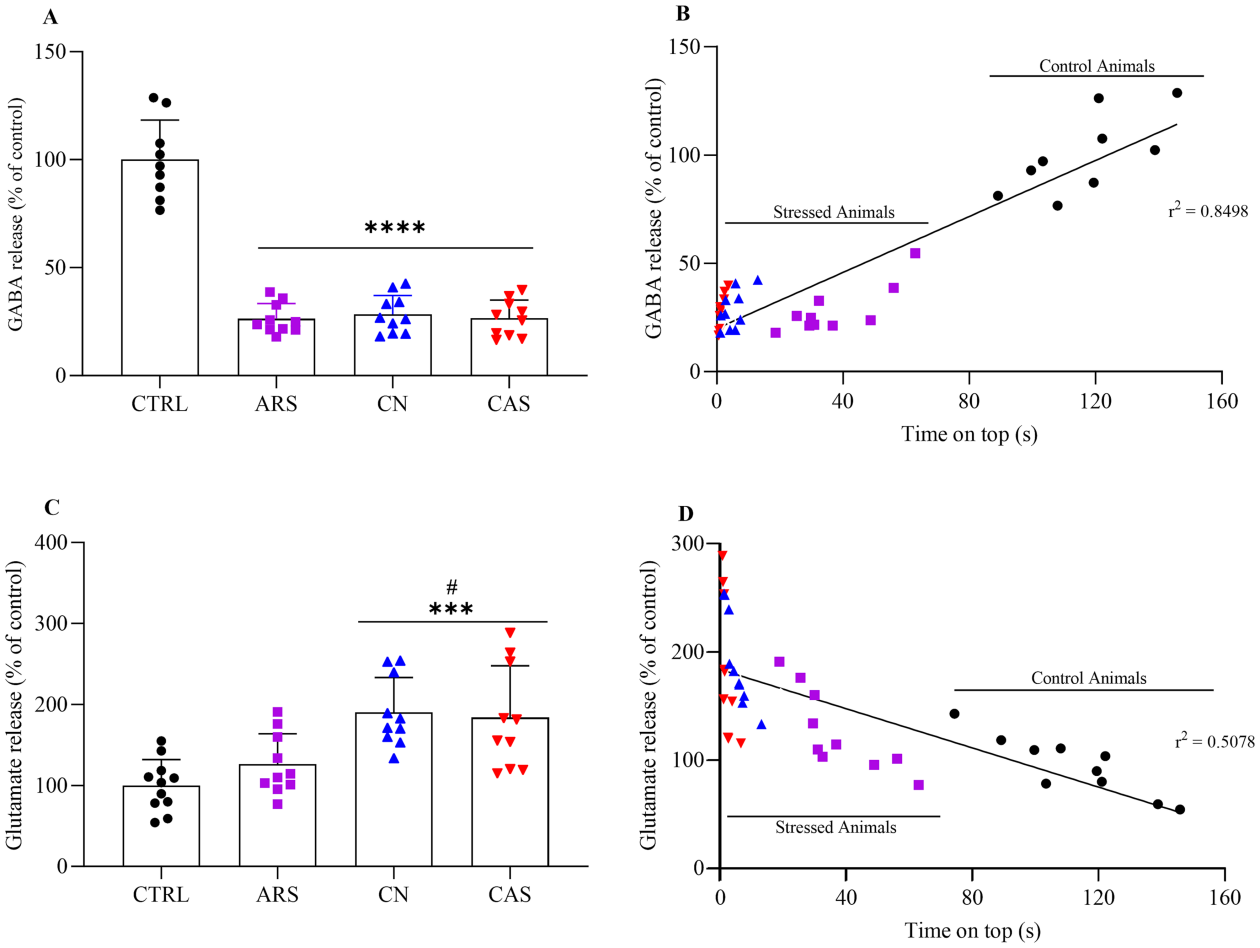
Extracellular levels of GABA (A) in the zebrafish brain submitted to ARS, CN and CAS with linear regression analysis between time spent on top and GABA release (B); glutamate release (C) and linear regression analysis between time spent on top and glutamate release (D) for each group. The values were expressed as percentage of control, compared using ANOVA one-way followed by Tukey *post hoc* test. ****p < 0.0001, ***p < 0.001, **p < 0.01, *p < 0.05. CTRL, control; ARS, Acute Restraint Stress; CN, Chasing with Net; CAS, Conspecific Alarm Substance. *vs. CTRL; # vs. ARS.

Neurochemical evaluations were performed in control and stressed zebrafish as demonstrated in Figure 1. Our results demonstrated that different stimuli elicit distinct behavioral responses within the spectrum of anxiety-like behavior. To explore these differences, we examined whether they were influenced by changes in the levels of the neurotransmitters GABA and glutamate, both of which play a modulatory role in the pathophysiology of this disorder. Our findings revealed that all aversive stimuli led to a reduction in GABA release by approximately 70% compared to the control group (F _(3, 35)_ = 96.03; *p* < 0.0001; Figure 4A). Furthermore, we analyzed the coefficients of determination to explore the relationship between the time spent in the top zone and neurotransmitter levels across groups. Our data indicate that high GABA levels are strongly correlated with increased exploration of the upper zone of the apparatus, as evidenced by r-squared value (r^2^ = 0.8498; *p* < 0.0001; Figure 4B). Additionally, our results showed that animals exposed to ARS did not exhibit changes in glutamate levels compared to the CTRL group (*p* = 0.3553; Figure 4C). In contrast, the CN and CAS groups showed an increase of approximately 74 and 68% in glutamate release compared to the CTRL groups, respectively (*F*_(3, 37)_ = 9.773; *p* < 0.0001; Figure 4C). In addition, we examined the coefficients of determination between time spent exploring the top of the apparatus and glutamate levels. Our data suggesting that higher glutamate levels may be linked to reduced exploration time at the top, as evidenced by R-squared value (r^2^ = 0.5078; *p* < 0.0001; Figure 4D). These findings suggest that the different behavioral responses observed following exposure to aversive stimuli may be correlated with specific changes in GABA and glutamate levels in the brains of these animals.

## 4 Discussion

In the present study, we observed for the first time that stress-induced anxiety behavior through different stressors exposure not only results in varying magnitudes of anxiety phenotypes but also elicits diverse neurochemical responses. Those results demonstrate the complexity of anxiety disorders, emphasizing that both behavioral and neurochemical outcomes are highly context dependent.

It is well known that in the Novel Tank Test, vertical locomotion is a parameter used to assess anxiety-like behavior due to the animal’s tendency to escape from the water surface (Kysil et al., 2017). Previous results from our group have already shown that when fish are exposed to Acute Restraint Stress there is a decrease in the time spent on top (Assad et al., 2020; Lucas Luz et al., 2021). Taken together, these studies suggest that increased exploration of the upper zone is related to reduced anxiogenic behavior, and remaining in the lower zone may reflect anxiety-like, depressive-like, or fear-like behavior. Our results align with this understanding, showing that animals exposed to acute stress exhibited a robust anxiogenic effect in the Novel Tank Test, including significantly less time spent at the top. However, despite the overall decrease in time spent at the top across all exposed groups, the animals exposed to the Alarm Substance and the group subjected to Chasing Net showed an even more pronounced reduction in this parameter. Previously, Egan et al. (2010) observed that exposure to the alarm substance acutely, but not chronically, was capable of inducing anxiety-like behavior in a robust manner, being observed by the reduction in time spent at the top, increase in freezing time and erratic movements.

Zebrafish, when faced with aversive conditions tend to use defense strategies that range from preference for safe environments at the bottom to increased freezing time and erratic movements during swimming (Kalueff et al., 2013). Therefore, the data from the present study confirm this understanding since the stressors used could induce an increase in freezing time. However, this effect was more pronounced in the groups exposed to physical or chemical stress stimuli compared to the group exposed to spatial restrictions or not exposed. Mongeau et al. (2003) propose that animals with a low level of anxiety tend to flee when faced with an aversive stimulus, while highly “anxious” animals freeze. Added to this, we also observed that when exposed to the stressors used here, the animals increased the frequency of erratic swimming. And interestingly, we again observed a differential behavioral response between the stressors, where the CN and CAS groups presented higher frequencies compared to the ARS, with high intensity in the CAS group. These factors appear to have been decisive in the impairment of locomotion in animals subjected only to physical and chemical stress, observed by the increase in the average speed covered by the animals. Furthermore, due to the increase in erratic movements, marked by unpredictable and rapid changes in swimming direction, these two groups (CN and CAS) presented a higher maximum speed, unlike animals controlled under stress due to spatial restriction. Together, these findings support the hypothesis that exposure to different stress stimuli induces varying magnitudes of anxiety-like behavior. Based on this, we hypothesize that this observed phenomenon is the result of the involvement of different brain regions and mechanisms that evaluate the nature and intensity of the stimulus to develop the specific behavioral response.

The amygdala represents the limbic structure involved in the generation of anxiety-like behavior induced by acute stress, where each nucleus is specialized in distinct neural connections (Tovote et al., 2015). In zebrafish, the subpallial amygdala is located in the regions of the ventral telencephalon (dorsal nucleus-Vd, supracommissural nucleus-*Vs* and post-commissural nucleus-Vp) associated with the mammalian central amygdala, possessing a larger population of GABAergic neurons (Ganz et al., 2012; Porter and Mueller, 2020), while the pallial amygdala is located in the dorsal telencephalon (medial dorsum-Dm and lateral dorsum-Dl), related to the mammalian basolateral amygdala, where the majority of neurons are glutamatergic (Wullimann and Mueller, 2004; von Trotha et al., 2014). It is well established that the GABAergic system plays a crucial role in anxiety disorders. Reduced levels of this neurotransmitter in the limbic system are often associated with diminished inhibitory control, which can lead to increased excitability and contribute to the development of the primary symptoms observed in anxiety disorders (Wierońska, 2011). Added to this, we observed a strong positive correlation between the time that animals remained at the top and the release of GABA. This finding suggests that animals that spent more time on top, reflecting less pronounced anxiety-like behavior, presented higher levels of GABA. In contrast, we demonstrated that only physical and chemical stressors were able to increase glutamate release in the zebrafish brain, which was not observed in the group exposed to the stressor due to spatial restriction. Thus, we observed a moderate negative correlation between time on top and glutamate release, where animals that exhibited more pronounced anxiety-like behaviors, such as those in CN and CAS groups, presented higher glutamate levels. These results highlight an inverse relationship between the inhibitory and excitatory systems that plays a crucial role in the regulation of stress responses, directly influencing the exploratory behavior and anxiety levels of the animals. In this sense, our data are linked to those found by Assad et al. (2020), who observed that acute restraint stress can inactivate the *Vs* subnucleus of the zebrafish telencephalon and consequently reducing GABA levels but does not alter the activation of the Dm and Dl regions, not being able to modulate the glutamate levels. These data suggest that the anxiety-like behavior induced by acute restraint stress was mediated only by GABAergic dysfunction. Thus, our hypothesis is that the low impact of the response to a spatially constrained stressor (ARS) may be related to its exclusive site of activation, while physical (CN) and chemical (CAS) stressors may depend on other pathways for perception of the stimulus.

In this regard, the Dm region receives sensory projections from the thalamus (somatosensory, visual, auditory) and the preglomerular complex (gustatory and odor) and sends projections to the subregions of the ventral nucleus and to the Periaqueductal Gray Matter (PAG; Northcutt, 2006; Lal and Kawakami, 2022). Calcium imaging studies show that the Dm region is activated in response to odors (Diaz-Verdugo et al., 2019; Bartoszek et al., 2021). Therefore, the increase of glutamate in the Dm subnucleus after a stressful stimulus can result in the activation of the PAG, which is specialized in the generation of defensive behavior, associated with more extreme defense reactions. This may be an important pathway in mediating anxiety-like behavior induced by chemical and physical stressors.

Despite physiological differences between zebrafish and humans, zebrafish exhibit anxiety-like behaviors in response to stressors that closely resemble those observed in mammalian and human models (Costa et al., 2023; Maximino, 2010). For instance, zebrafish exposed to physical and chemical stressors show anxiety-like behaviors like those observed in mice, indicating that these responses are governed by shared neurobiological mechanisms (De Abreu et al., 2014; Harada et al., 2024). This is further supported by studies demonstrating that stress-induced behaviors in zebrafish, such as reduced time spent in the upper zone of a tank are analogous to anxiety responses in other animal models (De Abreu et al., 2014; Kalueff et al., 2013). Our study advances this understanding by illustrating those different types of stressors—spatial, physical, or chemical—elicit varying magnitudes of behavioral and neurochemical responses in zebrafish. Specifically, our data show that distinct stressors activate different neurobiological pathways, as evidenced by variations in exploration time and neurotransmitter levels, including GABA and glutamate. These findings reflect the complexity of human anxiety disorders, as described in the DSM-V (American Psychiatric Association, 2013), where a range of stressors and anxiety manifestations are linked to intricate neurobiological mechanisms. The variations in GABA and glutamate levels in response to different stressors align with observations in mammalian models, where neurotransmitter systems are pivotal in regulating anxiety. This suggests that zebrafish can offer valuable insights into the neurochemical foundations of anxiety and support the development of more targeted pharmacological treatments. Understanding how different stressors affect these pathways can propose therapies tailored to the specific nature of the aversive stimulus.

Currently, the most effective anxiolytic medications are based on modulation of the GABAergic system, either by inducing an increase in the bioavailability of GABA or an increase in the affinity of the neurotransmitter to its GABAa receptor, such as benzodiazepines. However, due to the involvement of the glutamatergic system in the generation of anxiolytic behaviors, there is a need to carry out studies that aim to modulate this system with the aim of uncovering the mechanisms inherent to anxiety disorder, as well as improving the pharmacology currently used for treatment.

## 5 Conclusion

This study strongly suggests that different aspects of anxiety-like behavior are the result of differential neurochemical changes based on the action of GABA and, mainly, glutamate. That is, both behavioral and neurochemical results are highly context-dependent, supporting our initial hypothesis. Thus, we believe that the action of glutamate in anatomically distinct areas of the brain may be linked to different aspects of the anxiety response. Exploring this issue may open new pharmacological possibilities that consider the nature of the aversive stimulus and offer a deeper understanding of the pathogenesis of anxiety disorders induced by acute stress.

## Data Availability

The original contributions presented in the study are included in the article/supplementary material, further inquiries can be directed to the corresponding author.
